# Betalains: A Narrative Review on Pharmacological Mechanisms Supporting the Nutraceutical Potential Towards Health Benefits

**DOI:** 10.3390/foods13233909

**Published:** 2024-12-03

**Authors:** Renata M. Martinez, Cristina P. B. Melo, Ingrid C. Pinto, Soraia Mendes-Pierotti, Josiane A. Vignoli, Waldiceu A. Verri, Rubia Casagrande

**Affiliations:** 1 Department of Pharmaceutical Sciences, Health Sciences Center, Londrina State University, Londrina CEP 86039-440, Brazil; renatamimartinez@uel.br (R.M.M.); cristinadepaulamelo@gmail.com (C.P.B.M.); carol.ingrid2@gmail.com (I.C.P.); soraia.pierotti@uel.br (S.M.-P.); 2 Department of Biochemistry and Biotechnology, Centre of Exact Sciences, Londrina State University, Londrina CEP 86055-900, Brazil; javignoli@uel.br; 3 Department of Immunology, Parasitology and General Pathology, Biological Sciences Center, Londrina State University, Londrina CEP 86055-900, Brazil

**Keywords:** betacyanin, betaxanthin, antioxidant, anti-inflammatory, antihypertensive, hypolipidemic, antidiabetic, hepatoprotective, anticancer, antimicrobial

## Abstract

Betalains are naturally occurring pigments sourced mainly from *Beta vulgaris* (beetroot), *Hylocereus* spp. (dragon fruit), *Amaranthus* spp., and *Opuntia* spp. Betalains are widely used for their vibrant colors and health-promoting properties. These nitrogenous, water-soluble pigments are crucial colorants in the food industry, responsible for the red, purple, and yellow plant tissues, predominantly in the order Caryophyllales. They are grouped into betacyanins, with reddish-violet hues, and betaxanthins, yellow to orange. Examples include beetroot stems for betacyanins and yellow pitaya pulp for betaxanthins. Several pharmacological activities were reviewed in the scientific literature, describing their potential implications for human health. In this review, we focused on the main and latest studies on the pharmacological effects and mechanisms of betalains, including antioxidant, anti-inflammatory, antihypertensive, hypolipidemic, antidiabetic, hepatoprotective, neuroprotective, anticancer, and antimicrobial properties, in both in vitro and in vivo studies. Overall, betalain consumption is considered safe, with no major adverse effects or allergic reactions reported. We also approached topics such as the pharmacokinetics, bioavailability, stability, and enhanced stabilization of betalains. This article provides a comprehensive overview of bioactive potential of betalains, highlighting the biochemical mechanisms involved. The current knowledge broadens the clinical applicability of betalains, making them potential sources of nutraceutical compounds that can be used to develop functional foods.

## 1. Introduction

Betalains are the main compounds responsible for the red color of flowers, fruits, and other plant tissues, commonly found in plant groups in the order of Caryophyllales. These compounds have a positive impact on human health, in addition to their mercantile function as food additives and colorants [1,2]. These phytochemicals have a hydrophilic profile and are concentrated in cell vacuoles, attributing powerful coloration to a variety of plant components including leaves, fruits, roots, and other plant tissues [3].

One of the main species discussed in the literature concerning phytochemicals is *Beta vulgaris* from the Amaranthaceae group. The term betalains originates from the first identification of the tuberous root known as beet. Furthermore, the plant’s components, including betanin, which is the major element of the group extracted from red beet, have presented a therapeutic defense in inflammatory events, tumors, and metabolic syndrome [4].

A complementary source of natural colorants of the Amaranthaceae family comes from *Amaranthus tricolor*, especially containing betacyanins and betaxanthins with accentuated coloring capacity and antioxidant action [5]. Recently, new betalains were detected by a betalamic acid decarboxylated from dopamine in species of the Amaranthaceae family (beet, chard, celosia, and quinoa) employing mass spectrometry. The findings revealed the existence of new bioactive compounds primarily in *Chenopodium quinoa*, suggesting their contribution to the resistance of this species in the face of multiple environmental stressors [6]. Moreover, decarboxibetalains demonstrated potential usefulness in reducing oxidative stress and anti-aging effects in the *Caenorhabditis elegans* animal model, offering new perspectives on natural pigments to be explored further for health promotion [6,7].

This review aims to comprehensively discuss the potential characteristics of betalains and their biological activities. In addition to their use in the food manufacturing industry, it is essential to contextualize the efficacy of betalains in their applicability as valuable nutraceuticals. Amplifying their ability means harnessing the benefits of these natural metabolites acting directly on human health as supplementary therapy and disease prevention. The information on the betalains in question integrates significant updates, evidencing advances in their therapeutic purpose and the understanding of their biogenesis.

## 2. Chemical Structures of Betalains

**Figure 1:**
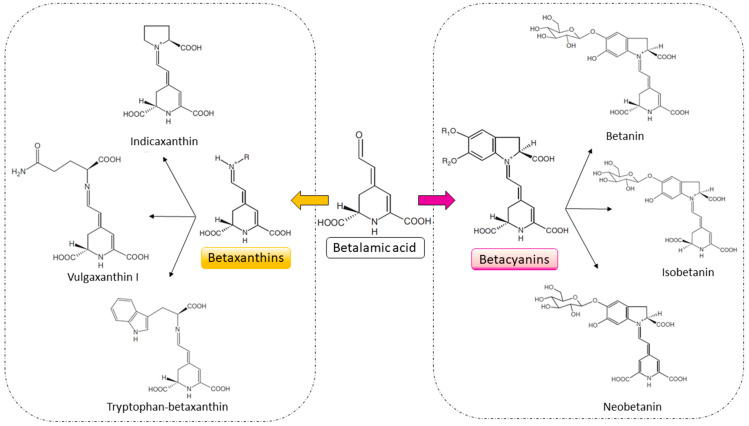
Chemical structures of the main subclasses of betalains. In the center, betalamic acid, the common precursor of betalains, gives rise to two main subclasses: betaxanthins (left, yellow/orange), which result from the conjugation of betalamic acid with amino acids or amines, and betacyanins (right, red/purple), formed by conjugation with cyclo-DOPA groups. Examples of betaxanthins include indicaxanthin, vulgaxanthin I, and tryptophan-betaxanthin, while examples of betacyanins include betanin, isobetanin, and neobetanin. The arrows indicate the biosynthetic pathways between betalamic acid and its derived subclasses. Adapted from Khan et al., 2015 [9].

Betalains are natural pigments belonging to the class of Schiff bases, responsible for the red, purple, and yellow coloration in various plants, especially in the Amaranthaceae and Cactaceae families. Chemically, these pigments are derived from betalamic acid, which is formed through the condensation of the amino acid tyrosine with dopaquinone, leading to the formation of a cyclic imine structure (Figure 1) [8]. Betalains are divided into two main groups: betacyanins (red/purple) and betaxanthins (yellow/orange). The structure of betacyanins includes a cyclic system derived from betalamic acid, associated with an indole-like ring, while betaxanthins are formed by the conjugation of betalamic acid with amines or amino acids [9]. The core structure of betalains is a highly conjugated system, which provides stability to the pigment and allows for light absorption in visible wavelengths, resulting in their vibrant colors [10]. These molecules are soluble in water, facilitating their use in the food and pharmaceutical industries as natural colorants. Nevertheless, their stability can be affected by factors such as light, pH, and temperature, which must be considered when formulating products [11].

## 3. Beneficial Health Effects of Betalains

### 3.1. Antioxidant

Betalain phytonutrients are pigments used in nutraceutical manufacturers because of their trace of coloration and natural origin. However, the applicability of these elements to human health has been investigated globally. One of the biological activities of growing importance is antioxidant activity [12]. Betalains are potent antioxidants and have demonstrated significant in vitro reactive oxygen species (ROS) scavenging properties (Table 1) [13]. Oxidative stress and inflammation are recognized as active components in the pathophysiology of various diseases. The exacerbated release of ROS destabilizes cellular machinery and redox homeostasis, as well as promoting genetic mutagenesis. As a result, consolidating the endogenous antioxidant function can restore cellular function and slow down the deleterious effects of oxidative stress [14,15,16]. The biological potential of beet as a functional food has been explored. Its antioxidant performance is attributed to its betalain components (betanin) and other phenolic compounds. The anti-free radical capacity of betanin is explained with its chemical structure containing sets of hydroxyls and unsaturations in the benzene ring. Betanin prevents oxidative damage to proteins by inhibiting the nitration of the amino acid tyrosine [17,18]. The suppression of free radicals mediated by the action of betalains present in *Opuntia* spp. extracts using the lipoxygenase fluorescein (LOX-FL) method was evidenced by blocking the action of lipoxygenase-1, an enzyme that, among other functions, acts in the generation of ROS such as peroxyl and hydroxyl [19]. In another work, the phytochemical profile and biological properties of the betalains found in the fruit of the species *Opuntia ficus-indica* (L.) Mill. were appreciated. The betalains indicaxanthin and betanin were identified as the most abundant compounds in the extracts of the plant. In addition, the research showed excellent antioxidant results in cactus pear peel extracts in assays evaluating oxygen radical absorbance capacity (ORAC), Ferric reducing antioxidant power (FRAP), a reduction of the 2,2-diphenyl-1-picrylhydrazyl radical (DPPH), and Trolox equivalent antioxidant capacity (TEAC) [20]. Red pitaya is also a powerful nutraceutical that contains a high grade of betacyanins, contributing to the reduction of oxidative damage induced by hydrogen peroxide in mouse embryonic fibroblast (3T3-L1) cells [21].

The presence of betalains was discovered in extracts of quinoa grains (*Chenopodium quinoa*) and was associated with an improvement in the antiradical capacity measured by the FRAP, ORAC, and ABTS [2,20-azino-bis (3-ethylbenzothiazoline-6-sulfonic acid)] assays in the grains of the plant [22]. In the last year, the potential activity of betalains has been tested in mice to combat brain oxidative damage induced by *Plasmodium berghei*. After the administration of betalains (from *Beta vulgaris*), concentrations of the antioxidants superoxide dismutase (SOD) and glutathione (GSH) were optimized and the lipid peroxidation marker malondialdehyde (MDA) was expressively reduced [23]. As an outcome, the data were aligned with a significant reduction in parasitemia, suggesting that betalains may block the multiplication of the protozoa by suppressing the intracellular transport of choline [9,23]. In short, the antioxidant potential of betalains has been revealed in different experimental research models, suggesting a surprising source in the context of metabolic disorders involving the production of ROS that deserves to be explored further.

### 3.2. Anti-Inflammatory

The inflammatory process is an endogenous physiopathological mechanism, which is essential for defense against pathogens but also to clear dead cells to allow tissue repair to occur. However, prolonged inflammation or auto-immune response leading to inflammation can become the disease itself [24]. A notable property of betalains described in the literature is the ability to restrict the inflammatory response (Table 2). The ultrasound-assisted extraction of *Opuntia stricta* var. Dillenii’s prickly pears resulted in extracts rich in betalains such as betanin, isobetanin, and neobetanin with anti-inflammatory action measured by the inhibition of hyaluronidase [25]. In an in vitro model of intestinal inflammation using cultured Caco-2 cells (a cell line derived from colorectal adenocarcinoma), three betalains (betanin, vulgaxanthin I, and indicaxanthin) presented a significant downregulation of inflammatory enzymes/molecules such as cyclooxygenase-2 (COX-2), inducible nitric oxide synthase (iNOS), and inflammatory cytokines IL-6 and IL-8 [26]. In a model of chronic inflammatory bowel disease (IBD), the incubation of a human intestinal epithelial cell line (Caco-2 cells) with indicaxanthin at nutritionally relevant levels prevented the IL-1β activation of NADPH oxidase (NOX-1) and the consequent generation of ROS, which activates nuclear factor-κB (NF-kB) and the release of inflammatory cytokines IL-6 and IL-8, PGE2 with an increase in ROS release in a dose-dependent manner. In cells stimulated with IL-1β and incubated with indicaxanthin, the activation of NOX-1 and NF-kB was prevented, and the expression of COX-2 and inducible NO synthase was reduced [27].

**Figure 2:**
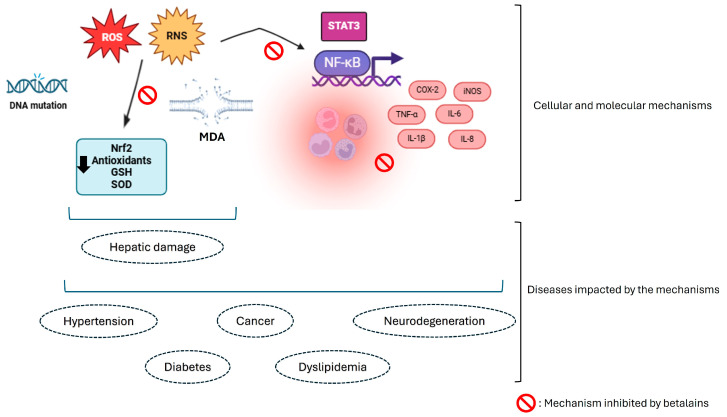
A representative diagram highlights oxidative stress and inflammation as participants in the pathophysiological mechanisms of diseases and the inhibition of these processes by betalains. The excessive generation of free radicals triggers important molecular alterations including genetic mutation, lipid peroxidation, and the impairment of the endogenous antioxidant system. In addition, it can activate inflammatory signaling routes, resulting in inflammatory components that aggravate the damage. ROS: reactive oxygen species. RNS: reactive nitrogen species. Nrf2: nuclear factor erythroid 2-related factor 2. GSH: glutathione. SOD: superoxide dismutase. MDA: malondialdehyde. STAT3: signal transducer and activator of transcription 3. NF-κB: nuclear transcription factor kappa B. COX-2: cyclooxygenase-2. iNOS: inducible nitric oxide synthase. TNF-α: tumor necrosis factor-alpha. IL: interleukin. Created using http://BioRender.com (accessed on 21 November 2024).

The biological activity of betalains was explored in an experimental cardiac injury model caused by isoproterenol subcutaneous injection in Wistar rats. Betalain pretreatment exerted a cardioprotective effect by limiting inflammation parameters such as the production of pro-inflammatory cytokines, interleukin (IL)-1β, tumor necrosis factor (TNF-α), IL-6, and chemokine receptor CXCR-4 (C-X-C chemokine receptor type 4) and the expression of the transcriptional regulator STAT3 (Signal transducer and activator of transcription 3) [28]. Studies from our laboratory analyzed the effectiveness of *Beta vulgaris* dye enriched in betalains in the in vivo model of inflammatory pain caused by carrageenan [15,29]. Our data demonstrated that betalain-rich dye reduced the production of inflammatory mediators TNF-α and IL-1β. In a second series of experiments, bone marrow-derived macrophages were incubated with betalains before lipopolysaccharide (LPS). LPS is an inflammatory stimulus that activates NF-κB and increases the levels of the cytokines IL-1β and TNF-α, while betalains limited the effects of LPS [29]. To conclude, in vivo and in vitro evidence demonstrates that betalains from varied sources can reduce inflammation. The main mechanism targeted by betalains is the transcription factor NFkB and antioxidant responses. As outcomes of these mechanisms of action, betalains minimize the production of cytokines, ROS, and reactive nitrogen species (RNS), thus reducing inflammation (Figure 2). Likewise, betalains exhibit potential in treating various diseases through their antioxidative and anti-inflammatory properties, primarily by modulating markers involved in oxidative stress and inflammatory responses.

### 3.3. Antihypertensive

Chronic arterial hypertension, a condition in which the blood pressure remains consistently high, strains the heart and damages blood vessels, increasing the risk of heart disease, stroke, and kidney failure [30]. Hypertension is typically characterized by the overactivation of the sympathetic nervous system (SNS) and the renin–angiotensin–aldosterone system (RAAS), leading to the excessive production of catecholamines, angiotensin II, and aldosterone, with consequent vasoconstriction, increased heart rate, cardiac hypertrophy, and sodium and water reabsorption [31]. Inflammation and oxidative stress also play active roles in the hypertension mechanism. Angiotensin II, aldosterone, and sodium-induced hypertonicity trigger the release of pro-inflammatory cytokines like TNFα, IL-1β, IL-6, IL-17, and IL-23, contributing to hypertension through inflammation [32]. Cytokines are linked to vascular fibrosis, endothelial dysfunction, and stiffness, which impair blood pressure regulation [33]. Additionally, oxidative stress driven by the increased production of reactive oxygen species (ROS) in endothelial cells, vascular smooth cells, neuronal cells, and renal tubular cells and enhanced by factors such as angiotensin II, high sodium, and catecholamines exacerbate inflammation and vasoconstriction [34].

There is a considerable number of studies focusing on the cardiovascular benefits of betalain-rich species. However, much of what is known about the benefits of beetroot juice to the cardiovascular system, including blood pressure control, is related to its particularly high nitrate level [35,36]. An elevated nitrate intake increases the organic production of nitric oxide through the nitrate–nitrite–NO synthase pathway. Inorganic nitrate originating from the diet, such as in beetroot juice, is considered a major active compound because nitric oxide is already well known for its cardio-metabolic effects, which include improving endothelial function and reducing blood pressure [37]. Dragon fruit and prickly pear do not contain significant amounts of nitrate but are rich in other bioactive compounds such as flavonoids, polyphenols, and vitamins, particularly vitamin C in prickly pear, which also have antioxidant and anti-inflammatory activities [38,39].

Because betalains are major compounds in beetroot juice and cactus pear, some clinical studies have addressed the potential of betalain-rich beetroot supplements to treat cardiovascular diseases, including hypertension. A pilot randomized crossover trial showed that betalain/betacyanin-rich supplements significantly lowered both systolic and diastolic blood pressures in male coronary artery disease (CAD) patients. This effect was associated with the decrease of atherosclerotic risk markers such as homocysteine, glucose, cholesterol, and triglycerides [40]. Data from a randomized, double-blind, placebo-controlled crossover trial investigating the effects of acute and short-term consumption of the betalain-rich, red-fleshed dragon fruit (*Hylocereus polyrhizus*) on the vascular function of young and healthy adults indicate that dragon fruit consumption has the potential to provide significative improvement in endothelial function and arterial stiffness, possibly due to its high betalain content. It was estimated that betalainic compounds correspond to about 85% of all active compounds in *Hylocereus polyrhizus* fruit [41]. The exact hypotensive mechanism of betalamic compounds is still not very well known. For instance, Tawa et al. [42] investigated whether betanin isolated from beetroot, at physiological concentrations, had an enhancing effect on vasorelaxation through different mechanisms using in vitro organ chamber techniques. The study concluded that acute exposure to betanin did not improve the vasorelaxant responses of the coronary artery to sodium nitrite and sodium nitroprusside and that the exposure of the coronary artery to betanin did not modulate the responses of two different endothelium-dependent vasorelaxants, bradykinin (receptor-dependent agonist) and A23187 (receptor-independent agonist). Because it is common knowledge that normal blood pressure depends on a balance between vasodilation and vasoconstriction, they also investigated betanin’s ability to inhibit vasoconstriction. It was demonstrated that betanin did not attenuate PGF2α and ET-1-induced vasocontraction. Therefore, the study suggests that betanin alone does not play a central role in the beetroot-induced acute lowering of blood pressure effect; however, because it contains a variety of active substances, the study concludes that the vasorelaxant effect observed in clinical essays may be due to the betanin synergistic effect of these substances [42]. Another in vivo study also demonstrated that acute i.v. betanin administration did not promote blood pressure reduction in rats, but promoted a transient increase instead [43]. On the same note, a study comparing beetroot and sodium nitrate to control hypertension in obese rats found similar functional and molecular responses to beetroot and sodium nitrate, suggesting that the nitrate content of beetroot was the factor responsible for inflammation reduction and cardiovascular improvement in rats with metabolic syndrome, rather than betanin [44].

Given the potent anti-inflammatory and antioxidant properties of betalains, it is reasonable to hypothesize that they may interfere with active inflammatory and oxidative mechanisms in hypertension and possess the potential to protect against hypertension-mediated tissue damage. As discussed previously in this article, various studies have demonstrated the anti-inflammatory and antioxidant effects of betalain-rich extracts and isolated betalain compounds in different experimental models. Betanin was also capable of modulating ROS generation and gene expression to activate antioxidant enzymes and reduce cytokine release, preventing endothelial damage and atherogenesis [45]. Furthermore, betanin and indicaxanthin isolated from *Opuntia ficus-indica* could bind to low-density lipoprotein (LDL) in vitro and were prominent in preventing copper-induced lipid oxidation. This antioxidative activity is believed to protect blood vessels, enhance nitric oxide bioavailability, and promote vasodilation. In this sense, long-term betalain intake could protect the cardiovascular system leading to reduced blood pressure [46]. Furthermore, human umbilical vein cord cells (HUVEC) were stimulated with human oxLDL (Oxidized low-density lipoproteins [oxLDL]—a model resembling hypercholesterolemia-related inflammation) and pre-treated with the phytochemical indicaxanthin from *Opuntia ficus indica*. The therapy attested to a defensive role in vascular dysfunction, being able to mitigate the expression of mRNA for inflammatory components such as intercellular adhesion molecule-1 (ICAM-1), vascular cell adhesion molecule-1 (VCAM-1), and endothelial leukocyte adhesion molecule-1 (ELAM-1) on a dose-dependent mode [47]. Another suggested anti-hypertensive mechanism is betalains’ ability to reduce peripheral vascular resistance by influencing the renin–angiotensin system (RAS) and inhibiting angiotensin-converting enzyme (ACE), promoting vasodilation and reducing blood pressure. For instance, betacyanins from beetroot juice have shown the ability to inhibit ACE, a key enzyme that regulates blood pressure [48].

In conclusion, the multifactorial nature of hypertension requires treatments that address its complex mechanisms, including the roles of the SNS, RAAS, inflammation, oxidative stress, and vascular dysfunction. It is confirmed by studies that the ingestion of a nutrient-rich diet or supplement capable of exerting synergistic positive effects would be much more efficient in controlling high blood pressure than the use of an individual antioxidant [49,50]. Betalains and betalain-rich extracts offer promising antihypertensive potential due to their antioxidant and anti-inflammatory properties. Clinical trials have shown that betalain-rich supplements can significantly lower blood pressure and improve vascular function, though their effects may be synergistic with other compounds, such as nitrates. While the exact mechanisms of betalains in blood pressure control are still being elucidated, their potential in reducing oxidative stress, cytokine production, and vascular damage highlights their role as a functional dietary approach for managing hypertension and promoting cardiovascular health (Table 3).

### 3.4. Hypolipidemic

Betalains have demonstrated promising activity in the management of dyslipidemia. These compounds have been shown to effectively lower total cholesterol (TC), triglyceride (TG), and low-density lipoprotein (LDL) levels while increasing high-density lipoprotein (HDL) levels in animal and human subjects. Furthermore, the modulation of lipid profiles through mechanisms like the reduction of fatty acid synthesis and enhancement of lipid mobilization have been observed following betalain treatment (Table 4). Their potential as a natural intervention for managing dyslipidemia makes them an exciting area of research for cardiovascular health improvement.

Studies in animal models clearly demonstrate the efficacy of betalains in lipid regulation. The oral administration of 300 mg/kg of extracts of *Hylocereus polyrhizus* (pitaya), containing high concentrations of betacyanin, significantly reduced TC and TG levels in hypercholesterolemic rats after 10 days of treatment [51]. Furthermore, in a hypercholesterolemic rat model, the extract of beetroot (*Beta vulgaris*) shows significant lipid metabolism benefits, reducing TC and TG while increasing HDL [52]. A four-week supplementation with beetroot crisps, rich in betalains, resulted in reductions in TC, TG, and glucose levels in dyslipidemic rat models, suggesting that the addition of beetroot crisps could alleviate metabolic changes [53]. Moreover, the consumption of pitaya (*Hylocereus polyrhizus*), rich in betalains, promoted a reduction in cholesterol (200 and 400 mg/kg) and an increase in HDL levels (400 mg/kg) in alloxan-induced diabetic mice [54]. The 21-day administration of an extract of *Amaranthus tricolor*, containing betalains (400 mg/kg), on alloxan mediated diabetes in rats, significantly reduced serum TG, TC, and LDL levels, and increased HDL level [55]. Betanin supplementation (25 and 100 mg/kg/day) was shown to mitigate isoproterenol-induced acute myocardial infarction in rats by improving cardiac structure and function. Mechanisms included the inhibition of LDL levels, inducible nitric oxide synthase (iNOS), NF-κB activation, and oxidative damage (such as SOD, MDA, catalase, and GSH) and ROS [56].

In cellular studies, betalains from *Opuntia stricta* var. dillenii fruit pulp effectively reduce triglyceride accumulation in 3T3-L1 mature adipocytes by decreasing fatty acid synthesis and increasing triglyceride mobilization, highlighting their potential health benefits in lipid regulation [57]. Three varieties of *Opuntia ficus-indica* rich in betalains show varying effectiveness in reducing the triglyceride accumulation in both 3T3-L1 maturing pre-adipocytes and mature adipocytes, highlighting the potential of these compounds in lipid regulation at the cellular level [58]. Furthermore, as discussed earlier in this review, betanin and indicaxanthin have demonstrated the ability to bind to LDL in vitro, effectively preventing copper-induced lipid oxidation [46]. Additionally, HUVECs, stimulated with human oxidized LDL (a model mimicking hypercholesterolemia-associated inflammation), were pre-treated with indicaxanthin derived from *Opuntia ficus-indica*. This treatment exhibited a protective effect by significantly reducing the mRNA expression of key inflammatory markers, supporting its potential anti-inflammatory role [47].

Data obtained with human subjects have shown promising results regarding the ability of betalains to improve lipid profiles. In a study involving 30 healthy subjects, consuming 250 mL of beetroot juice alongside a carbohydrate meal significantly reduced TC, TG, and LDL levels, highlighting beetroot’s potential lipid-lowering benefits due to its betalain content [59]. Physically active individuals (group of 30 infantry soldiers) in another study who received beetroot juice supplementation for 15 days experienced significant improvements in antioxidant status and plasma lipid profiles, with decreased LDL levels and increased HDL levels [60]. Additionally, a two-week regimen of betalain-/betacyanin-rich supplements, 50 mg/day, a betalain-rich supplement of red beetroot, or a betacyanin-rich supplement of *Opuntia stricta*, significantly decreased the levels of TC, TG, and LDL in the plasma of male patients with coronary artery disease [40].

In summary, the evidence supporting the hypolipidemic effects of betalains is promising, with preclinical and clinical studies showing significant improvements in lipid profiles. These findings suggest that betalains from sources such as *Hylocereus polyrhizus*, *Beta vulgaris*, *Amaranthus tricolor*, and *Opuntia* species have the potential to complement current dyslipidemia treatments. Furthermore, their antioxidant and anti-inflammatory mechanisms may contribute to broader metabolic benefits, including mitigating oxidative stress and inflammation-associated lipid metabolism dysregulation. However, further research is necessary to explore their long-term safety, optimal dosing, and mechanisms of action in humans to solidify betalains as viable nutraceutical options in the management of hyperlipidemia and related cardiovascular disorders.

### 3.5. Antidiabetic

Betalains, beyond their hypolipidemic properties, exhibit significant antidiabetic potential. Studies have demonstrated that betalains can effectively lower blood glucose levels, enhance insulin secretion, and reduce oxidative stress, positioning them as a valuable functional food component for diabetes control (Table 5). Their ability to inhibit critical enzymes such as α-amylase and α-glucosidase, which are involved in carbohydrate metabolism, makes them promising for blood glucose regulation [61,62].

In animal models, betalains have shown significant efficacy in glycemic control. Betanin (10–40 mg/kg/day) administered orally for 28 days alleviated oxidative stress through the Nrf2 signaling pathway in streptozotocin-induced diabetic rats’ livers [63]. The supplementation of dietary pigment betanin can offer an effective approach for the management of type 2 diabetes, as it has been demonstrated to ameliorate the effect of betanin by modulating hepatic carbohydrate metabolic enzyme activities and glycogen content in a diabetes rodent model caused by streptozotocin and nicotinamide administration, utilizing peripheral glucose and improving insulin secretion through the regeneration of pancreatic beta-cells [64]. The antidiabetic activity of *Opuntia matudae* fruit, in which betalains are present, has been observed in healthy mice, which presented a reduction of the blood glucose peak at the postprandial period and of hepatic glucose output in liver slices. In streptozotocin-induced diabetic mice, *Opuntia matudae* fruit extract reduced blood glucose levels [65]. Additionally, the administration of an aqueous extract of *Amaranthus tricolor* containing betalains at 200 and 400 mg/kg to diabetic rats reduced blood glucose levels in a dose-dependent manner, highlighting the antidiabetic potential of betalains [55]. A study found that 28 days of treatment with pitaya (*Hylocereus polyrhizus*), rich in betalains (200 mg/kg), significantly reduced blood glucose and lipid peroxidation in alloxan-induced diabetic mice [54].

The anti-diabetic activity of *Beta vulgaris* extract was determined by measuring inhibitions of α-amylase and α-glucosidase, as detected by in vitro analyses, with positive results also supported by in silico molecular docking [62]. Further, extracts from the leaves and inflorescences of *Amaranthus cruentus*, rich in betalains (betacyanin and betaxanthin), demonstrated benefits for diabetes management, because in vitro and in silico molecular they inhibited the enzymes α-amylase and α-glucosidase. Furthermore, in vivo tests on a normoglycemic murine model showed improved glucose homeostasis after sucrose load, which was significantly different from the control. When comparing the effects of the extracts to control drugs like glibenclamide and acarbose, the extracts demonstrated a moderate yet significant effect on blood glucose regulation. This suggests a milder action profile, which could be advantageous in various clinical scenarios [61].

Clinical studies have shown that the daily consumption of beetroot juice in patients with type 2 diabetes mellitus, for a 12-week period, reduced the concentrations of inflammatory markers, including IL-6, TNF-α, and NF-κB, which are involved in the pathogenesis of complications of type 2 diabetes [66]. Raw red beetroot consumption (100 g, daily), for 8 weeks in type 2 diabetes patients, resulted in a significant decrease in fasting blood sugar levels and glycosylated hemoglobin [67]. Moreover, four weeks of pitaya fruit consumption showed great potential and was beneficial in controlling the blood glucose level and lipid profiles of type 2 diabetic subjects [68]. *Opuntia ficus-indica* ingestion stimulated the peripheral disposal of oral glucose before and after exercise in healthy men [69]. Additionally, a two-week regimen of betalain-/betacyanin-rich supplements, 50 mg/day, a betalain-rich supplement of red beetroot, or a betacyanin-rich supplement of *Opuntia stricta* decreased the plasma fasting blood glucose of male patients with coronary artery disease [40].

In summary, preclinical and clinical findings suggest that betalain-rich foods exhibit promising anti-diabetic properties. These foods can help regulate blood glucose levels, reduce inflammatory markers, and modulate key carbohydrate-metabolizing enzymes, making them valuable in managing type 2 diabetes and associated metabolic disorders. Betalains positively affect the management of several metabolic syndrome risk factors, including hyperglycemia and dyslipidemia [70,71,72]. Therefore, foods rich in betalains possess substantial potential as functional interventions for enhancing metabolic health and mitigating hyperglycemia and dyslipidemia.

### 3.6. Hepatoprotective

The liver hosts various physiological processes, including the breakdown of foreign compounds. The metabolism of xenobiotics in the liver occurs in three phases intended to convert the molecules into more hydrophilic compounds facilitating their excretion. Phase I consists of oxidation, reduction, or hydrolysis to yield more polar metabolites and is promoted by cytochrome enzymes such as the cytochrome P450 (CYP450). Phase II occurs via conjugation reactions like glucuronidation or sulfation, along with phase III drug transporters, to promote the excretion of the metabolites. Even though these reactions are designed to detoxify xenobiotics into less toxic metabolites, they can sometimes produce reactive intermediates in the form of electrophilic molecules and excessive ROS, which can induce oxidative stress leading to liver cell toxicity and death [73,74]. This cellular toxicity unfolds through a cross-talk between oxidative stress and a complex network of events that includes damage to mitochondria, biochemical changes, the activation of immune cells, and the activation of apoptotic pathways [75]. Oxidative stress is an important event that can lead to the initiation and progression of liver damage promoted both by exogenous factors (e.g., alcohol, drugs, environmental toxins, chronic viral infection, radiation, and temperature) and by endogenous ones (e.g., obesity, insulin resistance) [76].

Krajka-Kuzniak et al. [77] demonstrated that betanin could protect human liver cells against ROS damage by upregulating the expression of phase II detoxifying enzymes including heme-oxygenase 1 (HO-1), NAD(P)H: quinone oxidoreductase 1 (NQO1), and glutathione S-transferases (GST) through the activation of the oxidative stress-sensitive nuclear factor (Nrf2) [77]. Betanin also increased the total antioxidant capacity in STZ-induced diabetic liver in rats, as well as decreasing lipoperoxidation (LPO) and enhancing the expression of Nrf2 and antioxidant enzymes such as superoxide dismutase (SOD) and glutathione peroxidases (GPx) [63].

Paraquat-induced liver damage is well known to involve mitochondrial damage and excessive ROS generation, in part due to the increased induction of CYPs 3A2 by paraquat. In a rat model of paraquat-induced liver damage, betanin antagonized the induction of CYP 3A2, suggesting a protective role for betanin, probably mediated by its antioxidant properties. In addition, betanin protected liver cells from ROS toxic effects when paraquat underwent redox cycling with the mitochondrial electron chain, damaging mitochondria and releasing pro-apoptotic factors [78]. Betanin also reduced carbon tetrachloride (CCl4)-induced liver toxicity in common carp (*Cyprinus carpio* L.) by inhibiting CYP2E1 activity and reducing oxidative stress [79]. Concerning the toxic effects of organophosphate pesticides on primary hepatocytes, betanin was observed to significantly enhance cell viability, markedly reduce ROS formation and LPO, replenish cellular GSH reserves, and protect mitochondria following treatment with chlorpyrifos, diazinon, and dichlorvos [80].

A study compared the hepatoprotective effect of *Opuntia robusta* fruit extract (OR, rich in betacyanins, betaxanthins, flavonoids, and other phenolic compounds), betanin, and N-acetylcysteine (NAC), which is the currently used treatment, against diclofenac-induced acute liver injury. The study demonstrated that OR and betanin exhibited hepatoprotective performance similar to NAC and that these results were achieved through iron chelation, prevention of lipid peroxidation, ROS scavenging, the restoration of GSH levels, the induction of NRf2 expression, and the prevention of apoptosis-mediated cell death [81]. Another study evaluated the activity of *Opuntia robusta* and *Opuntia streptacantha* fruit extracts against acetaminophen (APAP)-induced acute liver failure. In the study, betacyanins were identified as the main components of the *Opuntia* extracts, with betanin being the most abundant. The extracts significantly reduced biochemical, molecular, and histological markers of liver injury in both in vivo and in vitro models. This included reductions in markers such as AST, ALT, and LDH, indicating reduced liver damage. *Opuntia* extracts also reduced the expression of stress-related genes like Gadd45b. They influenced the expression of antioxidant-related genes (Sod2, Gclc, HO-1), suggesting that their protective effects were not solely due to their ROS-scavenging abilities. Gadd45b (Growth Arrest and DNA Damage-inducible Beta) is a member of the Gadd45 family of genes, which play a critical role in cellular responses to oxidative stress, inflammation, DNA damage, and cell cycle regulation. By lowering Gadd45b levels, the extracts potentially reduced the need for the liver cells to activate stress responses, implying a protective effect that minimized cellular damage and promoted survival without excessive stress signaling [82].

Experimental studies show that the hepatoprotective properties of betalains can be attributed to their capacity to reinforce cellular membranes, neutralize free radicals and electrophilic metabolites, and enhance the cellular redox balance by inducing the expression of antioxidant-responsive genes regulated by Nrf2, including the detoxifying enzymes heme-oxygenase 1 (HO-1), NAD(P)H: quinone oxidoreductase 1 (NQO1) and glutathione S-transferases (GST) [73,77].

In a pilot crossover clinical trial, patients with coronary artery disease received betalain-rich supplements derived from red beet and *Opuntia stricta*. The study showed that these supplements significantly increased SIRT1 levels, which are associated with longevity and cellular protection, and reduced markers of oxidative stress. Although the study focused primarily on cardiovascular benefits, the observed antioxidant effects may also suggest potential liver protection [83]. SIRT1 activators significantly reduced oxidative stress and inflammation by inhibiting the NF-κB and enhancing the Nrf2 pathway, contributing to cytoprotective effects [84]. The hepatoprotective properties of betalains, as demonstrated in numerous experimental studies, highlight their potential as natural antioxidants and detoxifying agents (Table 6). Among the betalains, betanin has shown an ability to protect liver cells from oxidative stress, to enhance antioxidant defenses by upregulating key enzymes such as HO-1, NQO1, and GST, and to mitigate cellular damage caused by toxins such as pesticides, paraquat, and acetaminophen.

To summarize, these protective effects of betalains are associated with the activation of the Nrf2 pathway, the inhibition of lipid peroxidation, and the restoration of redox balance. Although clinical studies on betalains are limited, existing studies suggest that betalain-rich supplements may have hepatoprotective effects, particularly against hepatic steatosis and oxidative stress. Further research is needed to fully understand the clinical efficacy of betalains in the prevention and treatment of liver disease. However, their promising antioxidant and anti-inflammatory properties underscore their potential as a therapeutic agent for liver protection.

### 3.7. Neuroprotection

Neuroinflammation and oxidative stress play significant roles in the progression of neurodegenerative diseases. The activation of glial cells, particularly microglia and astrocytes, contributes to chronic neuroinflammation. Microglia produces cytokines and neurotoxic factors such as ROS and nitric oxide in response to neuronal damage. Oxidative stress can activate inflammatory pathways, accelerating neuronal damage and disease progression [85]. Because betalains are widely recognized for their antioxidant and anti-inflammatory activities, they have been studied as compounds with great potential in the treatment of neurodegenerative diseases (Table 7).

Parkinson’s disease (PD) is characterized by the gradual loss of dopaminergic neurons in the substantia nigra pars compacta. Oxidative stress is identified as a critical component in the complicated progression of dopaminergic neuron death in all forms of PD [86]. A study evaluated the protective effects of betanin against oxidative damage and apoptosis induced by 6-hydroxydopamine (6-OHDA) and hydrogen peroxide (H_2_O_2_) in PC12 cells, a model for PD. Betanin significantly mitigated the reduction in cell viability caused by 6-OHDA and H_2_O_2_. It also decreased intracellular ROS levels suggesting antioxidant properties. 6-OHDA-induced apoptosis was markedly reduced by betanin, as determined by flow cytometry. Furthermore, betanin increased Survivin (an anti-apoptotic protein) levels and reduced Cyt c release (a pro-apoptotic protein). The decrease in the phosphorylation of SAPK/JNK, a pro-apoptotic signaling pathway, and the enhancement in the phosphorylation of PI3K supported cell survival [87]. Chronic neuroinflammation in PD is driven by pattern recognition receptors (PRRs), such as Toll-like receptors (TLRs). In PD, the upregulation of TLR4 on neuronal and glial cells initiates a signaling cascade that recruits the cytoplasmic adaptor molecule Myeloid Differentiation Primary Response-88 (MyD88) protein, generating the activation of NF-κB. This TLR/MyD88/NF-κB pathway accounts for the production of pro-inflammatory cytokines, such as TNF-α, IL-1β, and IL-6, contributing to neurodegeneration [88]. In a model of rotenone-induced PD in male Swiss albino mice, betanin dose-dependently reduced rotenone-induced movement impairments, levels of inflammatory cytokines (TNF-α, IL-1β, IL-6), and oxidative stress (elevated MDA and reduced GSH); it restored dopamine levels in the striatum and reduced neuronal degeneration. Furthermore, molecular docking indicated a strong binding affinity of betanin to NF-κB, suggesting that these effects appear to be mediated by targeting the TLR4/MyD88/NF-κB pathway [89].

Alzheimer’s Disease (AD) is characterized by the accumulation of amyloid-beta (Aβ) plaques and tau protein tangles, leading to synaptic dysfunction and neuronal death. AD characteristic neuroinflammation is triggered by microglial activation in response to Aβ plaques. Microglia release pro-inflammatory cytokines and ROS, contributing to chronic inflammation. Oxidative stress arises from mitochondrial dysfunction, impaired antioxidant defenses, and the pro-oxidative effects of Aβ. The ROS-generated damage of lipids, proteins, and DNA further amplifies neuronal death [90,91]. In a study using enhanced atomistic simulations to investigate the effects of astaxanthin, betanin, and epigallocatechin-3-gallate (EGCG) on Aβ peptide oligomers (dimer, trimer, tetramer), all three natural compounds inhibit Aβ aggregation by reducing aggregation-prone conformations. Betanin showed the most substantial modulation by significantly altering the secondary structure of Aβ oligomers: it increased β-sheet content at key regions and reduced aggregation-prone structures elsewhere, highlighting its potential as a therapeutic candidate for Alzheimer’s disease [92]. Betacyanin isolates and betacyanin-rich fractions were evaluated for anti-Aβ aggregation activity in human neuroblastoma (SH-SY5Y) cells using a thioflavin T fluorescence assay to measure the fluorescence intensity of Aβ aggregates. The study found that betacyanin-rich extracts from red pitaya exhibited significant anti-Aβ aggregation activity and that among the individual betacyanin isolates, hylocerenin showed the highest anti-Aβ aggregation activity, followed by phyllocactin and betanin. No neurotoxicity was detected by in vitro cytotoxicity assays [93].

High-fat diets (HFD) are linked to detrimental effects on brain health, manifesting through oxidative stress, neuroinflammation, mitochondrial dysfunction, impaired neurogenesis, and cognitive deficits [94]. Four weeks of oral treatments with indicaxanthin attenuated the neuronal damage effects of a ten-week high-fat diet in mice. Indicaxanthin reduced brain apoptosis by downregulating the expression of proapoptotic and upregulating antiapoptotic genes, reduced neuroinflammation by decreasing the expression of proinflammatory genes and proteins, and mitigated oxidative stress by reducing ROS and nitrogen species, malondialdehyde, and nitric oxide levels. The neuroprotective effects of indicaxanthin seem to derive primarily from its ability to regulate the redox-sensitive activation of the NF-κB and Nrf2 pathways and their associated downstream signaling mechanisms [95].

The progression of neurodegenerative diseases such as Parkinson’s disease (PD) and Alzheimer’s disease (AD) is driven by intricate interactions between oxidative stress and neuroinflammation. Oxidative stress triggers mitochondrial dysfunction and activates inflammatory pathways, including microglial activation and cytokine release. Betalains, particularly betanin and indicaxanthin, exhibit potent antioxidant and anti-inflammatory effects, targeting key mechanisms like ROS reduction, pro-inflammatory cytokine suppression, and the modulation of apoptosis-related pathways. Preclinical studies highlight their neuroprotective potential in PD by improving dopaminergic neuron survival and in AD by reducing Aβ aggregation and associated toxicity. Moreover, betalains’ ability to regulate NF-κB and Nrf2 pathways underscores their therapeutic promise in combating neurodegeneration induced by oxidative stress and chronic inflammation. These findings suggest that betalains and related natural compounds could serve as valuable adjunctive treatments in managing neurodegenerative diseases, warranting further investigation in clinical settings.

**Table 7:** Summary of neuroprotective mechanisms of betalains in various studies.

Betalains Source	Major Compound	Disease Model	Mechanisms	Reference
Betanin (isolated compound)	Betanin	PD model: 6-OHDA and H_2_O_2_–induced oxidative damage and apoptosis in PC12 cells	↓ ROS levels; modulation of SAPK/JNK and PI3K pathways to ↑ cell survival	[87]
Betanin (isolated compound)	Betanin	Rotenone-induced Parkinson-like mice model	↓ TNF-α, IL-1β, IL-6; ↓ MDA; ↑ GSH; ↓ expression of TLR4, MyD88, NF-κB	[89]
Betanin (isolated compounds)	Betanin	REMD simulations and Molecular Docking	↓ amyloid-beta aggregation	[92]
*Hylocereus polyrhizus*	betacyanins	Anti-A β aggregation activity and citotoxicity in SH-SY5Y cell culture	↓ amyloid-beta aggregation and no citotoxicity	[93]
Indicaxanthin (isolated compound)	Indicaxan-thin	High-fat diet-induced neuronal damage in mice	↓ expression of proapoptotic genes; ↑ expression of antiapoptotic genes; ↓ expression of neuroinflammatory genes and proteins; ↓ high-fat diet-induced oxidative stress	[95]

↓: decreased, ↑: increased.

### 3.8. Anticancer

Recent studies have shown that betalains exhibit anticancer properties and are being investigated for their potential use in cancer treatment (Table 8) [96,97,98]. A well-known example is betanin, a natural dye belonging to the betalain group. It is related to the induction of cancer cell apoptosis targeting mitochondria inducing the activation of caspases and the promotion of DNA fragmentation in cancer cells sparing normal cells [99]. The anticancer effects of betavulgarin, a compound isolated from beetroot, was explored specifically targeting breast cancer stem cells (BCSCs), a subpopulation known for drug resistance, self-renewal, recurrence, and metastasis. The results demonstrate that betavulgarin inhibits the proliferation, migration, and colony formation of breast cancer cells while also reducing mammosphere formation and the CD44+/CD24- subpopulation, which is linked to self-renewal. Additionally, betavulgarin decreases the levels of proteins like c-myelocytomatosis (C-Myc), Nanog, and octamer-binding transcription factor 4 (Oct4), essential for self-renewal, and inhibits the Stat3/Sox2 signaling pathway, which is vital for cancer stem cell survival [100].

Similarly, recent research compared the effects of beetroot juice from young shoots and mature roots on breast cancer cells (MCF-7 and MDA-MB-231). Juice from young shoots showed a stronger inhibitory effect on cancer cell proliferation, particularly in estrogen-dependent cells, and promoted both antiproliferative and apoptotic responses, pointing to the involvement of the internal apoptosis pathway [101]. The anti-tumor effects of betalains have also been investigated in an animal model using *Caenorhabditis elegans* JK1466 mutants, which develop germline teratomas due to the gld-1(q485) mutation. Researchers tested betalain-rich extracts and purified compounds, finding that tryptophan-betaxanthin reduced tumor size by 56.4% and increased lifespan by 9.3%. The antitumor activity was linked to the modulation of the DAF-16 transcription factor and the insulin signaling pathway [102]. The anticancer mechanisms of betalains include the modulation of cell signaling pathways such as the NF-kB pathway, the inhibition of angiogenesis, and the regulation of the cell cycle [101,103]. In addition, studies in murine models of skin and lung cancer have confirmed the ability of betalains to slow tumor growth and reduce metastasis [102,104].

A novel approach to treat cancer is to use nanocomposites of beet and chitosan; a study showed that the combination of the antioxidant properties of beet and the anti-proliferative effects of chitosan nanoparticles in a concentration of 250 μg/mL is effective in eliminating cancer cells, killing 89% of a human lung carcinoma (A549), 88% of a human ductal breast carcinoma (T-47D), and 83% of a human colorectal adenocarcinoma (Caco-2). The treatment acted by cellular apoptosis in all three cell lines with the expression of apoptotic markers, such as Caspase 3 protein (CasP3) and P53. Cellular necrosis was also induced [103,105]. In a colorectal cancer model (Caco-2 and HT-29), it was shown that the hydroalcoholic extract of beet (BHE) and betanin significantly inhibited the cancer cells’ growth in a dose- and time-dependent manner, with IC50 values ranging from 64 μg/mL to 107 μg/mL. Apoptosis analysis indicated an increase in the expression of pro-apoptotic genes (BAD, Caspase-3, Caspase-8, Caspase-9, Fas-R) and a reduction in the anti-apoptotic gene (Bcl-2) in the treated cells, suggesting that BHE and betanin suppress cancer cell proliferation and induce apoptosis by modifying gene expression [106]. Finally, the use of beet extract encapsulated in liposomes improved photodynamic therapy in the treatment of cancer. Molecular dynamics simulations carried out in this study showed that betanin interacts well with antiapoptotic proteins from the Bcl-2 family, suggesting that it can induce apoptosis in cells by inhibiting these proteins [104,107].

Collectively, these findings suggest that betalains have significant anticancer potential, demonstrating effects such as apoptosis induction, the inhibition of cancer cell proliferation, and the modulation of key signaling pathways like NF-kB and Stat3/Sox2. Innovative approaches, including betalain nanocomposites and liposomal encapsulation for photodynamic therapy, further enhance their therapeutic prospects. Overall, betalains represent a promising natural option for the development of novel cancer treatments.

**Table 8:** Summary of anticancer mechanisms of betalains in various studies.

Betalain Source	Major Compound	Disease Model	Mechanisms	Reference
Betanin (isolated compound)	Betanin	Cancer cells	Induction of apoptosis through mitochondria, activation of caspases, promotion of DNA fragmentation in cancer cells without affecting normal cells	[99]
*Beta vulgaris*	Betavulgarin	Breast cancer stem cells (BCSCs)	↓ proliferation, migration, colony formation, ↓ mammosphere formation, CD44+/CD24- subpopulation, and self-renewal proteins (C-Myc, Nanog, Oct4)	[100]
*Beta vulgaris* (young shoots/root)	Betalains	Breast cancer cells (MCF-7, MDA-MB-231)	↓ cancer cell proliferation, promoted antiproliferative and apoptotic responses, particularly in estrogen-dependent cells	[101]
Betalain-rich extracts and compounds	Tryptophan-betaxanthin	*Caenorhabditis elegans* JK1466 mutants	↓ tumor size by 56.4%, ↑ lifespan by 9.3%, modulated DAF-16 transcription factor and insulin signaling pathway	[102]
*Beta vulgaris* + Chitosan nanoparticles	Betalains and chitosan	Human lung (A549), breast (T-47D), colorectal (Caco-2) carcinomas	↓ 83–89% of cancer cells, induced apoptosis and necrosis, ↑ expression of apoptotic markers (Caspase 3, P53)	[103,105]
*Beta vulgaris* (hydroalcoholic extract)	Betanin	Colorectal cancer (Caco-2, HT-29)	↓ cancer cell growth (IC_50_ 64–107 μg/mL), ↑ expression of pro-apoptotic genes (BAD, Caspase-3, Caspase-8, Caspase-9, Fas-R), ↓ Bcl-2	[106]
*Beta vulgaris* (encapsulated in liposomes)	Betanin	Photodynamic therapy for cancer	Enhanced photosynthetic effect, improved stability and effectiveness, interaction with Bcl-2 proteins to induce apoptosis	[104,107]

↓: decreased, ↑: increased, C-Myc: c-myelocytomatosis, Oct4: octamer-binding transcription factor 4.

### 3.9. Antimicrobial

Betalains demonstrate significant antimicrobial activity against a wide range of pathogens (Table 9). Studies have demonstrated that betanin and isobetanin exhibit bactericidal effects against Gram-positive and Gram-negative bacteria, such as *Escherichia coli*, *Staphylococcus aureus*, and *Pseudomonas aeruginosa*. These effects are suggested to be due to betalains’ ability to interfere with the integrity of the bacterial cell membrane, resulting in cell lysis [108]. In addition to their direct action on membranes, betalains can generate reactive oxygen species (ROS) inside microbial cells, inducing oxidative stress and DNA damage, which contributes to their antimicrobial activity. The ability of betalains extracted from red beetroot to inhibit biofilm formation, particularly in *Candida albicans*, has also been investigated, exhibiting efficacy in reducing the adherence and viability of fungal cells [109]. The topical use of betalain-rich extracts has been suggested for the treatment of mild skin infections, although more research is needed to validate this clinical application [9]. In addition, due to their antimicrobial properties, betalains have been explored as natural preservatives in food products, extending shelf life by inhibiting microbial growth [110].

Overall, while betalains have demonstrated promising antimicrobial activity against a variety of pathogens, further preclinical in vivo studies are necessary to validate their efficacy and safety. Future research should explore the potential of the dual benefits of betalains, their anti-inflammatory and antimicrobial effects, particularly in treating infections. Additionally, investigating the pharmacokinetics of betalains is crucial to determine whether they accumulate in specific organs. For instance, certain antibiotics are more effective for urinary tract infections because that is where they accumulate. Whether the pharmacokinetics of betalains favors such possibility is unknown. These studies will provide a clearer understanding of the clinical relevance and potential applications of betalains as antimicrobial agents.

## 4. Pharmacokinetics, Bioavailability and Toxicity

Although betalains have exhibited evidence of biological efficacy in vivo, their bioavailability is considered to be very low, which could potentially limit their therapeutic potential [111]. In general, pharmacokinetic studies on betalains are based on the analysis of their concentration in body fluids after oral ingestion of betalain-rich foods or extracts, which are complex matrixes that contain a great variety of substances [111]. In this sense, it is important to consider that the synergistic or antagonistic interactions between the substances that constitute the natural matrix may influence key factors like absorption, metabolism, or excretion, which ultimately could impact the bioavailability of betalains [112].

Studies on human subjects have shown that cactus pear is a bioavailable source of betanin and indicaxanthin (Table 9). After ingesting 500 g of cactus pear pulp, both compounds peaked in plasma concentration after 3 h. Indicaxanthin had a longer plasma half-life (2.36 h) compared to betanin (0.94 h) and a much higher urinary excretion (76% vs. 3.7%). Both compounds were undetectable in plasma after 12 h [113]. Furthermore, in a study involving healthy volunteers (*n* = 8), the distribution of betanin and indicaxanthin in red blood cells (RBCs) after ingesting cactus pear fruit was analyzed. Indicaxanthin peaked in RBCs at 1.03 ± 0.2 μM at 3 h. A much lower concentration of betanin (0.03 ± 0.005 μM) was detected in RBCs 3 h after ingestion. Indicaxanthin remained in RBCs longer than betanin, being reduced by half (0.55 ± 0.06 μM) after 5 h and significantly by 8 h. Both pigments were demonstrated to increase RBCs’ resistance to oxidative damage and were undetectable after 12 h [114].

An in vitro study using Caco-2 cell monolayers showed that both indicaxanthin and betanin are absorbed through the intestinal epithelium but via different mechanisms. Indicaxanthin follows a non-polarized route without relying on membrane transporters, while betanin is limited by MRP2-mediated efflux, which reduces its absorption. Indicaxanthin’s absorption is more efficient, and its bioavailability is higher. The food matrix did not affect indicaxanthin’s absorption, but betanin’s absorption from beetroot was lower compared to cactus pear [115]. A human study found that only 0.5–0.9% of betanin was excreted in urine after the consumption of red beet juice, supporting the idea of limited absorption due to the food matrix, as shown in a prior study on cactus pear [116].

The availability of betacyanins present in fermented red dragon fruit drink (RDFD) and pressed red dragon fruit juice (RDFJ) was accessed in simulated gastric and intestinal digestion. Results showed that after being subjected to intestinal digestion, RDFD retained 46.42% of betanin while RDFJ retained 43.76%, with the impact of intestinal digestion being greater than the stomach environment [117]. The long-term intake (6 weeks) of fermented beetroot juice by human volunteers showed that during juice consumption, native compounds and twelve betalain metabolites (decarboxylated and dehydrogenated) were found in the blood and urine of volunteers. Betalains peaked in plasma after the first week of juice intake (87.65 ± 15.71 nmol/L) and in urine after the second week of juice intake (1.14 ± 0.12 μmol/L) (Table 10). It was also proposed that betalains would go through sequential biotransformation before and after absorption under physiological conditions [118].

The profile of betacyanins in the gastric content, blood, and urine of rats was evaluated and the results showed that they underwent degradation and absorption in the stomach in a dose-dependent manner [119]. Wang et al. [111] compared betacyanin excretion in humans through renal and intestinal routes after 14 days of red beetroot juice consumption. The juice was consumed with white bread to extend gastric retention time. The study confirmed previous findings of low urinary excretion rates and the limited bioavailability of betacyanins. It was observed that a large proportion of betacyanins underwent fragmentation, including deglucosidation and decarboxylation, in the gastrointestinal tract. A positive correlation between urinary and intestinal excretion indicated that betacyanin bioavailability is mainly regulated by gut biotransformation. Furthermore, the involvement of diverse gut bacteria demonstrated that intestinal transformation is more complex and subject to individual variation compared to systemic metabolism [111].

Reports indicate that betalains are safe for human consumption and non-toxic. Betaine has been classified by the European Union food additive code E-162 as safe for use in food, cosmetics, and pharmaceuticals and is particularly employed in foods that require the use of clean and safe ingredients [120]. Similarly, betaxanthins have been approved for use as food colorants; however, their use is limited by their low chemical stability [70].

A toxicological and toxicokinetic study of the oral administration of betalains from the fermented fruit of garambullo (*Myrtillocactus geometrizans*) in male and female Wistar rats showed that betalains were not acutely toxic up to the maximum dose (5 g/Kg of body weight) and were safely metabolized and excreted without side effects [121]. Another study on Wistar rats showed that the oral administration of betalain-rich Rivina berry (*Rivina humilis*) juice was safe up to 5 g/Kg. There were no signs of acute or subacute toxicity and no abnormalities in organ function, biochemical markers, or histological structure over 35 days [122]. Betalains extracted from red pitaya (*Hylocereus polyrhizus*) peel showed no toxicity after oral administration of up to 48,500 mg/kg body weight in mice [123]. Furthermore, betanin did not stimulate and even inhibited IgE and IgG, demonstrating the lack of allergic response to the pigment [9].

Betalains are biologically effective but their low bioavailability limits their therapeutic potential. Factors such as food matrix interactions, absorption mechanisms, and biotransformation processes in the gut play a crucial role in modulating betalain absorption and bioavailability. Continued research on betalain pharmacokinetics is essential for optimizing their use in therapies. Despite these limitations, betalains have consistently been found to be safe for human consumption, and toxicological studies in animals have shown no acute or subacute toxicity.

## 5. Stability and Enhanced Stabilization

The instability of betalains remains a significant limitation to their broader application in food and pharmaceutical products [124]. Several factors negatively impact betalain stability, including elevated temperature, light, oxygen, extreme pHs, metal ions, and high-water activity. Thermal degradation is a major challenge for betalain-based products, with betacyanins and betaxanthins losing stability at temperatures above 60 °C and 40 °C, respectively. At elevated temperatures, betalains, such as betacyanins, degrade into products like betalamic acid, neobetacyanins, and newly formed betaxanthins. The boiling of betanin-containing material evokes an increase in the percentage contribution of dehydrogenated and decarboxylated betanin derivatives, accompanied by elimination of the glycoside [9]. Betacyanins exhibit greater stability during storage at room temperature [8,125]. In terms of pH, betalains are generally stable within the 3 to 7 range. Betacyanins are most stable under acidic conditions, whereas betaxanthins are more stable at neutral pH [126,127]. In alkaline conditions, the aldimine bond hydrolyzes, breaking the conjugated system and leading to a loss of color intensity, while acidification causes the recondensation of betalamic acid with the amine group of the substituent residue [9].

Metal ions can catalyze oxidation, leading to color loss [125]. Oxygen exposure can also cause color changes from red/purple to orange/yellow. Oxidative stress caused by light, metals, or oxygen accelerates degradation pathways. The oxidative stability of betacyanins can be improved by further glycosylation [9]. Moreover, betalains exhibit the highest stability in low-humidity conditions, particularly when the water activity is below 0.63 [8,124,126]. The main challenge for betalains’ application in the food and pharmaceutical industries is their instability in response to these environmental factors. Therefore, stabilization and controlled release methods are frequently researched. Encapsulation and adsorption techniques are promising alternatives to overcome these limitations and improve the stability of bioactive compounds [128]. For example, betalains derived from cactus were successfully encapsulated using ionic gelation with calcium alginate, resulting in superior pigment stability and antioxidant activity compared to freeze-drying. After 25 days at 50 °C under various relative humidity conditions, there was a significant retention of betanin and indicaxanthin levels. Ionic gelation effectively protected betalains, making it a promising method for their use as functional colorants in the food industry [129]. Similarly, the adsorption of betaxanthin onto the macroporous resin LH-17 was highly effective, with dynamic column experiments demonstrating its potential for industrial separation, resulting in a 72.4% increase in purity compared to yellow beet extracts [130].

Thus, encapsulation and adsorption techniques can significantly enhance the stabilization and recovery of betalains, leading to increased efficiency and broader application possibilities. However, different wall materials and encapsulation methods have both benefits and limitations, which require careful consideration to optimize betalain stability while minimizing color alterations [110]. The extent of the impact of encapsulation and adsorption on the biological activities of betalains must also be investigated.

## 6. Conclusions

**Figure 3:**
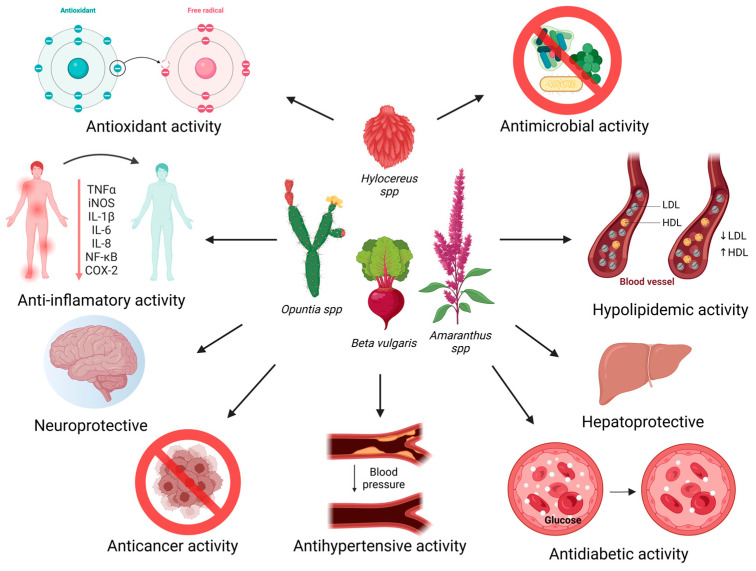
Representative scheme of the main biological properties and plant sources of betalains. Created using https://BioRender.com (accessed on 30 October 2024).

In conclusion, the beneficial functionality of many vegetables can be justified by the occurrence of natural metabolites including betalains. Our review emphasizes several studies demonstrating the substantial capacity of these pigments through pharmacological/nutraceutical mechanisms involved in the antioxidant response, anti-inflammatory, the control of dyslipidemia, glycemic regulation, the maintenance of cardiovascular health, liver protection, neuroprotection, and antitumor and antimicrobial activities (Figure 3). The varied bioactivities of betalains are predominantly linked to their capacity to modulate oxidative stress and inflammatory responses [15,29]. Thus, betalains hold significant potential for applications in the food, pharmaceutical, and cosmetic industries, particularly as therapeutic agents for maintaining health and curing diseases. To establish their reliability as nutraceutical agents, future studies must focus on comprehensive clinical trials to evaluate their pharmacokinetics, long-term safety, and optimal dosing. Investigating synergistic effects with other bioactive compounds and elucidating precise mechanisms of action in humans is also critical. Furthermore, enhancing the stability of betalains or developing advanced pharmaceutical formulations to modulate their release are promising strategies to achieve viable and effective products.

## Figures and Tables

**Table 1:** Summary of antioxidant effects of betalains in various studies.

Betalains Source	Major Compound	Model	Mechanisms	Reference
*Beta vulgaris*	Betanin	UV/Vis spectral analysis of peroxynitrite scavenging activity	↓ ONOO-	[18]
*Opuntia* spp.	Betalains	Lipoxygenase fluorescein (LOX-FL) method	↓ LOX-1	[19]
*Opuntia ficus-indica*	Indicaxanthin and betanin	In vitro antioxidant profile	↑ ORAC, ↑ FRAP, ↓ DPPH, ↑ TEAC	[20]
*Hylocereus polyrhizus*	Betacyanins	Mouse embryonic fibroblast (3T3-L1) cells	↑ FRAP, ↓ DPPH	[21]
*Chenopodium quinoa*	Betalains	In vitro antioxidant power of quinoa grains	↑ FRAP, ↑ ORAC, ↓ ABTS	[22]
*Beta vulgaris*	Betalains	Mice infected with *Plasmodium berghei*	↑ SOD, ↑ GSH, ↓ MDA	[23]

↓: decreased, ↑: increased, ONOO-: peroxynitrite, LOX-1: lipoxygenase-1, ORAC: oxygen radical absorbance capacity, FRAP: ferric reducing antioxidant power, DPPH: 2,2-diphenyl-1-picrylhydrazyl radical, TEAC: trolox equivalent antioxidant capacity, SOD: superoxide dismutase, GSH: glutathione, MDA: malondialdehyde.

**Table 2:** Summary of anti-inflammatory mechanisms of betalains in various studies.

Betalains Source	Major Compound	Model	Mechanisms	Reference
*Opuntia stricta*	Betanin, isobetanin, and neobetanin	Anti-inflammatory in vitro biological activities	↓ Hyaluronidase	[25]
*Beta vulgaris* and *Opuntia ficus-indica*	Betanin, vulgaxanthin I, and indicaxanthin	Model of intestinal inflammation using cultured Caco-2 cells	↓ COX-2, ↓ iNOS, ↓ IL-6, ↓ IL-8	[26]
*Opuntia ficus-indica*	Indicaxanthin	Human intestinal epithelial cell line	↓ NOX-1, ↓ COX-2, ↓ iNOS, ↓ IL-6, ↓ IL-8, ↓ PGE2	[27]
Betalain (isolated compound)	Betalain	Cardiac injury model caused by isoproterenol in Wistar rats	↓ IL-1β, ↓ TNF-α, ↓ IL-6, ↓ CXCR-4, ↓ STAT3	[28]
*Beta vulgaris*	Betalains	Hyperalgesia with carrageenan and bone marrow- derived macrophages	↓ TNF-α, ↓ IL-1β, ↓ NF-κB	[29]

↓: decreased, COX-2: cyclooxygenase-2, iNOS: inducible nitric oxide synthase, IL: interleukin, NOX-1: NADPH oxidase, PGE2: Prostaglandin E2, TNF-α: tumor necrosis factor-alpha, CXCR-4: C-X-C chemokine receptor type 4, STAT3: Signal Transducer and activator of transcription 3, NF-κB: nuclear factor kappa B.

**Table 3:** Summary of antihypertensive effects of betalains in various studies.

Betalain Source	Major Compound	Disease Model	Mechanisms	References
*Beta vulgaris* and *Opuntia stricta*	Betalain and betacyanin	Human trial with atherosclerotic cardiovascular disease patients	↓ SBP, ↓ DBP, ↓ atherosclerotic risk factors	[40]
*Hylocereus* *polyrhizus*	Betalains	Human trial with young and healthy adults	Improved cardiovascular function	[41]
Betanin (isolated compound)	Betanin	Isolated porcine arteries	Betanin alone did not improve vasorelaxation	[42]
*Beta vulgaris*	Beetroot juice	Rats received beetroot extract	Transient increase in blood pressure and heart rate	[43]
*Beta vulgaris*	Beetroot juice × sodium nitrate	Hypertensive and obese rats	↓ SBP, improved cardiovascular and metabolic function similar in both treatments	[44]
Betanin and Indicaxanthin (isolated compounds)	Betanin and indicaxanthin	LDL isolated from healthy volunteers’ plasma	↓ LDL cooper- induced oxidation, ↓ atherogenesis	[46]
*Opuntia fícus-* *indica*	Betanin	oxLDL stimulated HUVEC	↓ expression (ICAM-1, VCAM-1, and ELAM-1)	[47]
*Beta vulgaris*	Betacyanins and betaxanthins	In vitro ACE- inhibitory activity	Promotes vasodilation and reduces blood pressure by ↓ ACE	[48]

↓: decreased, SBP: systolic blood pressure, DBP: diastolic blood pressure, HUVEC: human umbilical vein cord cells, oxLDL: oxidized low-density lipoprotein, ICAM-1: intercellular adhesion molecule-1, VCAM-1: vascular cell adhesion molecule-1, ELAM-1: endothelial leukocyte adhesion molecule-1, LDL: low-density lipoprotein, ACE: angiotensin-converting enzyme.

**Table 4:** Summary of hypolipidemic effects of betalains in various studies.

Betalains Source	Major Compound	Disease Model	Mechanisms	Reference
*Hylocereus* *polyrhizus*	Betacyanin	Hypercholesterolemic rats	↓ TC, ↓ TG	[51]
*Beta vulgaris*	Betalains	Hypercholesterolemic rats	↓ TC, ↓ TG, ↑ HDL	[52]
*Beta vulgaris*	Betalains	Dyslipidemic rats	↓ TC, ↓ TG	[53]
*Hylocereus* *polyrhizus*	Betalains	Alloxan-induced diabetic mice	↓ TC, ↑ HDL	[54]
*Amaranthus* *tricolor*	Betalains	Alloxan-induced diabetic rats	↓ TC, ↓ TG, ↓ LDL, ↑ HDL	[55]
Betanin (isolated compound)	Betanin	Isoproterenol-induced acute myocardial infarction in rats	↓ LDL, ↓ iNOS, ↓ NF-κB, ↓ ROS	[56]
*Opuntia stricta*	Betalains	3T3-L1 mature adipocytes	↓ TG accumulation, ↓ fatty acid synthesis, ↑ TG mobilization	[57]
*Opuntia ficus*	Betalains	3T3-L1 maturing pre-adipocytes and mature adipocytes	↓ TG accumulation,	[58]
*Beta vulgaris*	Betalains	Healthy subjects consuming a carbohydrate rich meal	↓ TC, ↓ TG, ↓ LDL	[59]
*Beta vulgaris*	Betalains	Physically active human individuals	Improvements in antioxidant status, ↓ LDL, ↑ HDL	[60]
*Opuntia stricta* and *Beta vulgaris*	Betacyanin/Betalain	Male patients with coronary artery disease	↓ TC, ↓ TG, ↓ LDL	[40]

↓: decreased, ↑: increased, TC: total cholesterol, TG: triglycerides, LDL: low-density lipoprotein, HDL: high-density lipoprotein, iNOS: inducible nitric oxide synthase, NF-κB: nuclear factor kappa B, ROS: reactive oxygen species.

**Table 5:** Summary of antidiabetic mechanisms of betalains in various studies.

Betalains Source	Major Compound	Disease Model	Mechanisms	Reference
Betanin (isolated compound)	Betanin	Streptozotocin-induced diabetic rats’ livers	↓ oxidative stress through the Nrf2 signaling pathway	[63]
Betanin (isolated compound)	Betanin	Streptozotocin–nicotinamide induced rats	Modulated hepatic carbohydrate metabolic enzymes, ↑ Insulin secretion	[64]
*Opuntia matudae*	Betalains	Streptozotocin-induced diabetic mice	↓ Blood glucose levels	[65]
*Amaranthus tricolor*	Betalains	Alloxan-induced diabetic rats	↓ Blood glucose levels, ↓ lipid peroxidation	[55]
*Hylocereus polyrhizus*	Betalains	Alloxan-induced diabetic mice	↓ Blood glucose levels	[54]
*Beta vulgaris*	Betalains	In vitro and in silico analyses	↓ α-amylase and α-glucosidase	[62]
*Amaranthus cruentus*	Betacyanin and betaxanthin	In vitro, in silico analyses and normoglycemic murine model	↓ α-amylase and α-glucosidase (in vitro and in silico), improved glucose homeostasis (murine model)	[61]
*Beta vulgaris*	Betalains	Type 2 diabetes patients	↓ Inflammatory markers (IL-6, TNF-α, and NF-κB)	[66]
*Beta vulgaris*	Betalains	Type 2 diabetes patients	↓ Fasting blood sugar, ↓ glycosylated hemoglobin	[67]
*Hylocereus polyrhizus*	Betalains	Type 2 diabetic patients	Controlled blood glucose and improved lipid profiles	[68]
*Opuntia ficus*	Not *specified*	Healthy men	Stimulated peripheral glucose disposal before and after exercise	[69]
*Opuntia stricta* and *Beta vulgaris*	Betacyanin/Betalain	Male patients with coronary artery disease	↓ Plasma fasting blood glucose	[40]

↓: decreased, ↑: increased.

**Table 6:** Summary of hepatoprotective mechanisms of betalains in various studies.

Betalains Source	Major Compound	Disease Model	Mechanisms	Reference
Betanin (isolated compound)	Betanin	Human liver cells (in vitro)	↑ Nrf2 activation and ↑ detoxifying enzymes (HO-1, NQO1, GST)	[77]
Betanin (isolated compound)	Betanin	STZ-induced diabetic rat liver	↑ total antioxidant capacity; ↓ lipid peroxidation; ↑ Nrf2, SOD, GPx	[63]
Betanin (isolated compound)	Betanin	Paraquat-induced oxidative damage in rat liver	↓ ROS, ↓ CYP3A2 induction, and protection of mitochondria from redox cycling damage.	[78]
Betanin (isolated compound)	Betanin	Carbon tetrachloride (CCl4)-induced liver toxicity in carp	Inhibition of CYP2E1 activity, ↓ oxidative stress, and protection against liver toxicity	[79]
Betanin (isolated compound)	Betanin	OP toxicity on primary rat hepatocyte culture	↓ ROS, ↓ lipid peroxidation, ↑ GSH, protects mitochondria	[80]
*Opuntia robusta*	Betacyanins	Diclofenac-induced acute liver injury in rats	Iron chelation, ROS scavenging, ↓ lipid peroxidation, ↑ GSH, Nrf2 activation.	[81]
*Opuntia robusta/* *Opuntia streptacantha*	Betanin	Acetaminophen (APAP)-induced acute liver failure in rats	↓ AST, ALT, LDH, ↓ Gadd45b expression with upregulation of antioxidant genes (Sod2, Gclc, HO-1).	[82]
*Opuntia stricta*	Betalain-rich extract	Coronary artery disease in humans	↑ SIRT1 levels, ↓ oxidative stress, with potential liver protection through antioxidant pathways.	[83]

↓: decreased, ↑: increased, HO-1: Heme oxygenase 1, NQO1: NAD(P)H: quinone oxidoreductase 1, GST: Glutathione S-transferases, ACE: angiotensin-converting enzyme, SOD: Superoxide dismutase, GPx: Glutathione peroxidase, CYP3A2: Cytochrome P450 3A2, OP: organophosphates; CYP2E1: Cytochrome P450 2E1, ROS: Reactive oxygen species, GSH: Glutathione, AST: Aspartate aminotransferase, ALT: Alanine aminotransferase, Gadd45b: Growth Arrest and DNA Damage-inducible Beta, Sod2: Superoxide dismutase 2 (mitochondrial), Gclc: Glutamate-cysteine ligase catalytic subunit, SIRT1: Sirtuin 1.

**Table 9:** Summary of antimicrobial effects of betalains in various studies.

Betalains Source	Major Compound	Disease Model	Mechanisms	Reference
Betanin (isolated compound)	Betanin	Microbial cells	Generation of reactive oxygen species (ROS), inducing oxidative stress and DNA damage	[96]
Red beetroot extract	Betalains	*Candida albicans* (biofilm formation)	Inhibited biofilm formation, ↓ adherence and viability of fungal cells	[109]
Betalain-rich extracts	Betalains	Mild skin infections	Suggested for topical use, though further research needed	[9]
Betalains (various sources)	Betalains	Microbial growth in food products	Explored as natural preservatives to extend shelf life by ↓ microbial growth	[110]

↓: decreased.

**Table 10:** Summary of betalains pharmacokinetics.

Betalain Source	Major Compound	Model	Absorption Data	Half-Life data	Metabolism/ Excretion Data	Reference
Cactus pear	Betanin	Human	Plasma peak at 3 h.	0.94 h	Total clearance: 12 h after intake—3.7% eliminated in the urine.	[113]
	Indicaxanthin	Human	Plasma peak at 3 h.	2.36 h	Total clearance: 12 h after intake—76% eliminated in the urine.	[113]
Cactus pear	Betanin	Human (RBC)	Plasma peak at 3 h (0.03 ± 0.005 μM).	nd	Undetectable after 12 h.	[114]
	Indicaxanthin	Human (RBC)	Plasma peak at 3 h (1.03 ± 0.2 μM).	5 h (0.55 ± 0.06 μM)	Undetectable after 12 h	[114]
Beetroot/ cactus pear	Betanin	Trans-epithelial transport in Caco-2 cell monolayer	Absorption is limited by MRP2-mediated efflux.	na	na	[115]
Beetroot/ cactus pear	Indicaxanthin	Trans-epithelial transport in Caco-2 cell monolayer	Follows a non-polarized route without relying on membrane transporters.	na	na	[115]
Fermented red beet juice	Betalains	Human	Plasma peak after the first week (87.65 ± 15.71 nmol/L).	np	Urine peak after the second week (1.14 ± 0.12 μmol).	[118]

RBC: red blood cells; Caco-2: human epithelial colorectal adenocarcinoma cell line; MRP2: Multidrug Resistance-associated Protein 2; nd: not detected; np: not provided; na: not applicable.

## Data Availability

No new data were created in this study since it is a narrative review All discussed information can be found in the cited references. Data sharing is not applicable to this article.
